# Mechanisms underlying immune tolerance caused by recombinant *Echinococcus granulosus* antigens Eg mMDH and Eg10 in dendritic cells

**DOI:** 10.1371/journal.pone.0204868

**Published:** 2018-09-27

**Authors:** Yana Wang, Shiyu Lv, Qiang Wang, Chan Wang, Mingxing Zhu, Zhanbing Ma, Wei Zhao

**Affiliations:** 1 Department of Cell Biology and Medical Genetics of Schoool of Basic Medical Science, Ningxia Medical University, Yinchuan, Ningxia, China; 2 Echinococcosis Laboratory, Ningxia Medical University, Yinchuan, Ningxia, China; 3 Clinical Laboratory, Qianfoshan Hospital of Shandong Province, Jinan, Shandong, China; Auburn University College of Veterinary Medicine, UNITED STATES

## Abstract

Mice immunized with recombinant *Echinococcus granulosus* antigens Eg10 and Eg mMDH do not show elevated resistance to *E*. *granulosus* infection but show aggravated infection instead. To gain a deeper insight in the immune tolerance mechanisms in mice immunized with Eg10 and Eg mMDH, this study simulated the immune tolerance process *in vitro* by culturing bone marrow-derived dendritic cells (BMDCs) in the presence of Eg10 or Eg mMDH. Scanning electron microscopy revealed that Eg10- and Eg mMDH-treated DCs exhibited immature cell morphology, while addition of LPS to the cells induced changes in cell morphology and an increase in the number of cell-surface protrusions. This observation was consistent with the increased expression of the cell-surface molecules MHCII and CD80 in Eg10- and Eg mMDH-treated DCs pretreated with LPS. DCs exposed to the two antigens had a very weak ability to induce T-cell proliferation, but could promote the formation of Treg cells. Introduction of the indoleamine 2,3-dioxygenase (IDO) inhibitor, 1-methyl tryptopha (1-MT) enhanced the ability of the antigens to induce T cells and inhibited the induction of Treg cells. Eg mMDH-treated DCs showed a strong response to 1-MT: the DCs had high mRNA levels of IDO, IL-6, and IL-10, while 1-MT decreased the expression. In contrast, DCs treated with Eg10 did not show significant changes after 1-MT treatment. Eg mMDH inhibited DC maturation and promoted IDO expression, which, on the one hand, decreased the ability of DCs to induce T-cell proliferation, resulting in T-cell anergy, and on the other hand, induced the formation of Tregs, resulting in an immunosuppressive effect. In contrast, the escape mechanisms induced by Eg10 did not primarily depend on the IDO pathway and might involve other mechanisms that need to be further explored.

## Introduction

The tapeworm *Echinococcus granulosus* is a parasite that prevails in areas with developed animal husbandry and causes chronic infection, severely threatening human and animal health. After entering the human or animal (intermediate host) body, the oncosphere of *E*. *granulosus* can migrate to organs such as the liver, kidneys, lungs, and brain, where they develop to protoscoleces and form cysts, which can cause severe pathological organ damage that can even lead to death.

Studies have shown that after the worm enters the host, it is not removed by the immune system of the host but instead inhabits the host and gradually leads to chronic infection. The whole infection process likely involves two primary parts. One is *E*. *granulosus* forms cysts that are enveloped with a protective sheath, which allows the parasite to efficiently avoid immune cell attacks [1]. The other is some molecules of *E*. *granulosus*, such as antigen B (AgB) and hydatid fluid (HF), can tweak the host immunoreaction to generate immune escape or immune tolerance in favor of survival of the parasite [2]. The whole process involves complex immune-response mechanisms, in which many types of immune cells, such as dendritic cells (DCs), T cells, and B cells, and cytokines IL-4, IFN-γ, and IL-10 play a role, and studies have shown a direct relation between the immune escape of *E*. *granulosus* and a Th1/Th2 shift in the host [3].

After entry in the body, *E*. *granulosus* is first detected and captured by antigen presenting cells (APCs). To date, dendritic cells (DCs) are the only known professional APCs able to effectively activate T lymphocytes. DCs are widely present and play an essential role in balancing immune activation and immune tolerance [4]. DCs sense pathogens via receptors that recognize pathogen-associated molecular patterns so that they can activate specific signal pathways to initiate biological and immunological effects. DCs interact with other cells in the immune system and respond to specific antigens via intercellular cytokine interactions. Recent studies have shown that differences in the numbers, phenotypes, and functions of DCs can promote the occurrence of disease [5, 6]. Different antigens may stimulate DCs to differentiate into different subsets, which may further induce or participate in different immune response reactions [7]. DCs also play a pivotal role in the mechanisms of the parasite to induce immune tolerance through highly expression IDO. IDO is the only rate-limiting enzyme that exists outside the liver, and catalyzes the catabolism of tryptophan via the kynurenine pathway [8]. Studies have shown that IDO is involved in the immune escape of tumors, autoimmune disorders, and systemic inflammatory reactions, and high IDO expression can not only inhibit T-cell immunity but also induce the activation of Treg, playing an important role in the mechanisms of peripheral immune tolerance and immune escape [9, 10].

A preliminary animal experiment conducted in our research group revealed that the antigens Eg mMDH and Eg10 of *E*. *granulosus* had good antigenicity and immunogenicity [11,12]. However, mice immunized with Eg mMDH and Eg10 did not show an increased capability to resist reinfection by *E*. *granulosus*, but rather showed aggravated infection compared with the control group. The underlying mechanisms remained unclear. In this study, we conducted *in-vitro* culture of BMDCs with Eg10 and Eg mMDH to simulate the conditions in immunized mice. Using this system, we observed the morphological and functional changes of DCs as well as the expression of various cytokines and IDO in an effort to explore the immune tolerance mechanism of mice immunized with Eg10 and Eg- mMDH.

## Materials and methods

### Materials

#### Animals

The Institutional Review Board of Ningxia Medical University approved the study protocol, the approval number is 2018–226. C57BL/6 mice were bred in a specific pathogen-free facility from the Laboratory Animal Center of Ningxia Medical College.

#### *E*. *granulosus* antigens

Antigens Eg mMDH and Eg10 were expressed in the prokaryotic expression systems Eg mMDH/PET28a/BL21 and Eg10/PET28a/BL21, respectively, and existed as inclusion bodies in *Escherichia coli*. They were purified by Ni^2+^-affinity column chromatography and then subjected to refolding with a protein refolding solution, followed by 10-min centrifugation at 300 ×g and collection of the supernatant. The protein samples were processed with Triton X-114, and then, endotoxin was measured using a ToxinSensor^TM^ Chromogenic LAL Endotoxin Assay Kit (GenScript Inc. USA) according to the manufacturer’s instructions to make sure that the endotoxin concentrations were <0.02 EU/ml to avoid interference in subsequent experiments. The purified protein was detected with 12% sodium dodecyl sulfate-polyacrylamide gel (SDS-PAGE), which showed bands at 30 kD and 38 kD for Eg 10 and Eg mMDH respectively. These proteins were sterilized by filtration through 0.45-μm filter membranes (MILLEX-HP; Millipore) and quantified with a protein assay kit (Bradford China), then, aliquoted and stored at –20°C for later use.

#### BMDC culture

Two C57BL/6 male mice of 6–8 weeks of age were killed by neck fracture and soaked in 75% ethanol for 5–10 min. Then, the femur and tibia of the two posterior limbs were collected. After the tissues were removed from the bones, the metaphysis was exposed and repeatedly rinsed with RPMI-1640(Gibco, invitrogen) culture medium using a 25G needle. Bone marrow cells were collected, suspended, and mixed with erythrocyte lysate buffer (Solarbio, China) at 4°C at a 1:4 ratio. The mixture was centrifuged at 300 × g, after which the supernatant was discarded. The pelleted cells were resuspended in complete culture medium containing 10% fetal bovine serum (FBS) (Hyclone laboratories, Inc.), 2 mM glutamine (Sigma-Aldrich), 50 μM β-mercaptoethanol (Sigma-Aldrich), 1% penicillin and streptomycin (Gibco invitrogen), 10 ng /ml GM-CSF, and 5 ng /ml IL-4 (R&D Systems, Abingdon, United Kingdom) at a concentration of 2×10^6^ /ml. The cell suspension was added to 12-well plates (Corning Incorporated, USA) (2 ml/well) for culture at 37°C under 5% CO_2_. At 3 and 5 days after the onset of culture, half of the culture was replenished with fresh culture medium to supplement GM-CSF and IL-4 cytokines. At 7 days, non-adherent cells were collected for counting. Then, the cells were suspended in complete culture medium without GM-CSF and IL-4 at a concentration of 1×10^6^ cells/ml and inoculated in 24-well plates.

#### Treatments

In experiment (1), DCs were incubated with Eg mMDH (1 μg/ml), Eg10 (1 μg/ml), or lipopolysaccharide (LPS) (100 ng/ml Escherichiacoli strain 0111:B4; Sigma-Aldrich) in RPMI-1640 culture medium in 24-well plates for 20 h. Next, LPS was added to the Eg mMDH- or Eg10-treated DCs, while Eg10 or Eg mMDH were added to the LPS-treated DCs, after which the cells were incubated for another 18 h. Non-treated DCs in RPMI-1640 culture medium were used as a negative control, and LPS-treated DCs were used as a positive control.

In experiment (2), DCs were treated for 3 h with tryptophan (250 mΜ Sigma-Aldrich), a substrate for IDO, in 24-well plates in the presence or absence of 1 mM 1-MT (Sigma-Aldrich), an inhibitor of IDO. Next, Eg mMDH (1 μg/ml), Eg10 (1 μg/ml), LPS (100 ng/ml), IFN-γ (10 ng/ml eBioscience), or RPMI-1640 culture medium were added and the cells were cultured for another 20 h. Cells cultured in RPMI-1640 culture medium alone were used as a negative control, and DCs treated with IFN-γ were used as a positive control for IDO.

### Methods

#### Determination of the phagocytic abilities of DCs

BMDCs of each treatment group in experiment (1) were collected, and fluorescein isothiocyanate(FITC)-dextran (1 mg/ml, 40kDa, Sigma-Aldrich) was added a final concentration of 1 mg/ml. The mixture was incubated at 37°C or 4°C (for control binding) for 1 h and then centrifuged, followed by the addition of 500 μl 1% paraformaldehyde (PFA). Then, the content of FITC-labeled dextran phagocytosed by BMDCs was measured by flow cytometry (Guava plus, USA). Each sample was analyzed in triplicate, and data were collected for 10,000 cells. FITC fluorescence intensities were indicative of the phagocytic abilities.

#### T-cell purification

Wild-type C57BL/6 mice of 6–8 weeks of age were killed by cervical dislocation. Under sterile condition the spleens were gained and grinded, filtered through 200-mesh sieves to collect spleen cells, to which erythrocyte lysate buffer was added. The mixture was centrifuged for 10 min, followed by cell isolation using CD4^+^ beads (Miltenyi Biotec) according to the manual. Carboxyfluorescein diacetate succinimidyl ester (CFSE 2·5 μM Sigma) was added to the CD4^+^ T per 1×10^7^ cells at a final concentration of 2.5–5 μM. Then, the cells were incubated in the dark at 37°C for 15 min, after which the reactions in the culture were terminated with pre-cooled FBS. The cells were rinsed twice with 10% FBS in RPMI-1640 medium and finally diluted to the desired concentration. The entire process was conducted in the dark.

#### Mixed lymphocyte culture

To BMDCs of each treatment group, mitomycin C (25 μg ml^−1^ Merck) was added at a ratio of one mitomycin C molecule per 2×10^5^ cells, at a final concentration of 25 μg/ml, and the cells were incubated at 37°C for 30 min. These BMDCs were used as stimulator cells and were incubated with CD4^+^ T cells (used as effector cells) at BMDC: CD4^+^ T-cell mixing ratios of 1:10, 1:20, and 1:50. Three duplicate wells with 200 μl of culture medium without stimulator cells were set as control wells. The cells were cultured for 5 days, after which the cells were analyzed by flow cytometry. The fluorescence intensities were used to evaluate the proliferative abilities of T cells.

#### Scanning electron microscopy

DCs stimulated with Eg mMDH, Eg10, LPS and RPMI 1640 were cultured on coverslips 2h, fixed in 2.5% glutaraldehyde for 20 min and washed 3 times PBS, then stored overnight at 4°C. Cells were then routinely osmicated and dehydrated. Samples were incubated in tert-butyl alcohol, vacuum dried, sputter-coated with gold, and examined under a scanning electron microscope (SEM, S-3400N, Hitachi, Japan). Selecting one hundred cells from every group randomly count DC cell with dendrites or without and compare them. (If a cell has dendrites less than 3 counted as without dendrite). Then, choose 20 cells with dendrites and count, analyze the number of dendrites in each cell.

#### Flow-cytometric detection of DC-surface molecules

BMDCs of each treatment group in experiment (1) were collected and were divided into two tubes, to which anti-mouse MHCII-PE-(yanin5-conjugated) Cy5, anti-mouse CD80-FITC, and anti-mouse CD11c-PE (phycoerythrin), anti-mouse CD86-FITC, and anti-CD40-PE-cy5 (eBioscience), respectively, were added, followed by the addition of 500 μl of 2% PFA in PBS, pH 7·4. A blank control was included. All samples were incubated in the dark at 4°C for 30 min, and were then subjected to flow-cytometry detection of surface molecules of BMDCs. Data were collected for at least 1×10^4^ cells, and the experiment was carried out four times.

#### Detection of the kynurenine content in the culture supernatant by ELISA

Culture supernatant of each BMDCs treatment group in experiment (2) was collected and the kynurenine content in the supernatant was measured by ELISA using a kynurenine-3-monooxygenase kit (Shanghai jianglai biotechnology lnc, China) as per the manual. Three duplicate wells were set for each sample. Absorbance is read at 450 nm.

#### Detection of the IDO content in DCs by fluorescence immunohistochemistry

Eg mMDH-, Eg10-, LPS-, and RPMI-1640-treated DCs (2×10^5^ cells) were collected and stained with CFSE, grow on the cell climbing slides. After 20-h incubation, the slides were removed from the culture medium and rinsed twice with PBS, followed by fixation of the cells with 4% PFA for 20 min and treatment with 3% H_2_O_2_ for 20 min. Then, the cells were incubated with sheep serum (blocking solution, Boster biological lnc. China) for 30 min and incubated overnight at 4°C with mouse IDO polyclonal antibody (Millipore) that had been pre-diluted at a ratio of 1:100. Next, the cells were rinsed three times with PBS and iFluor647 goat anti-mouse antibody (AAT Bioquest USA) was added. After 30-min incubation at room temperature, the cells were rinsed with PBS, and observed under a fluorescence microscope (Olympus Japan). For each treatment group, 100 DCs were randomly selected and DCs labeled with green and red double fluorescence were expressing IDO and DCs labeled with only green fluorescence were not expressing IDO, and these cells were counted for statistical comparison.

#### Evaluation of mRNA expression by quantitative (q) PCR

Each group of DCs as elaborated in the above grouping section (2) was collected, and extract total RNA (Trizol Invitrogen). The cDNA products were obtained with the RevertAid First Strand cDNA Synthesis Kit (Thermo). The content of IDO, IL-6, TNF-α, IL-2and IL-10 mRNA in each group of DCs was detected by real time qPCR (Bio-rad USA). The experiment was performed in triplicate and the mean values were reported.

IDO: up primer: 5'-AGCAATCCCCACTGTATCCA-3';
down primer: 5'-GGTCCACAAAGTCACGCATC-3' 131bp
TNF-**α**up primer: 5'- ACTGGCAGAAGAGGCACTCCC-3';
down primer: 5'-GGCTACAGGCTTGTCACTCGAATT-3' 247bp
IL-6: up primer: 5'-CCACTTCACAAGTCGGAGGCTTA-3';
down primer 5'-GCAAGTGCATCATCGTTGTTCATAC-3' 112bp
IL-10: up primer: 5'-TGGACAACATACTGCTAACCGA-3';
down primer: 5'-ACCCAGGGAATTCAAATGC-3' 158bp
IL-2: up primer: 5'-CAACAGCGCACCCACTTCAAG-3'
down primer: 5'-CCGCAGAGGTCCAAGTTCATCTT-3' 242bp
β-actin: up primer: 5'- CATCCGTAAAGACCTCTATGCCAAC-3';
down primer: 5'- ATGGAGCCACCGATCCACA-3' 171bp

#### Detection of cytokines of culture supernatant

Level of tumor necrosis factor alpha (TNF-α), IL-2, IL-6 and IL-10 of different groups in culture supernatants collected at 20 h were detected by enzyme-linked immunosorbent assays (ELISA) (kits; MultiScienceS Biotech Co., China) according to the manufacturer’s instructions. Three duplicate wells were set for each sample. Absorbance is read at 570nm and 630nm.

#### Detection of CD4^+^CD25^+^Foxp3^+^ Tregs

DCs from each treatment group in experiment (2) were collected and incubated with T cells at a ratio of 1:10 for 96 h according to the aforementioned method for mixed lymphocyte culture. Then, the cells were stained with the Mouse Regulatory T Cell Staining Kit #3 (eBioscience) as instructed in the manual. DCs (1x10^6^ cell/tube) were stained with surface antigens CD4 FITC at 0.125 μg and CD25 PE at 0.06 μg. Add 1 ml of 1X Permeabilization working solution to each tube and incubate for 30 minutes at room temperature. Centrifuge samples at 300 x g for 5 minutes at room temperature, then discard the supernatant. Add Foxp3 PE-Cy5antibody 0.5 μg/tube to cells and incubate for 30 minutes at room temperature in the dark and isotype control are as same method. Resuspend stained cells in an appropriate volume of Flow Cytometry Staining Buffer and measured samples on a flow cytometer.

The data were collected for at least 1×10^4^ cells in each sample.

## Results

### Expression of DC-surface molecules upon interaction with *E*. *granulosus* antigens

**Fig 1:**
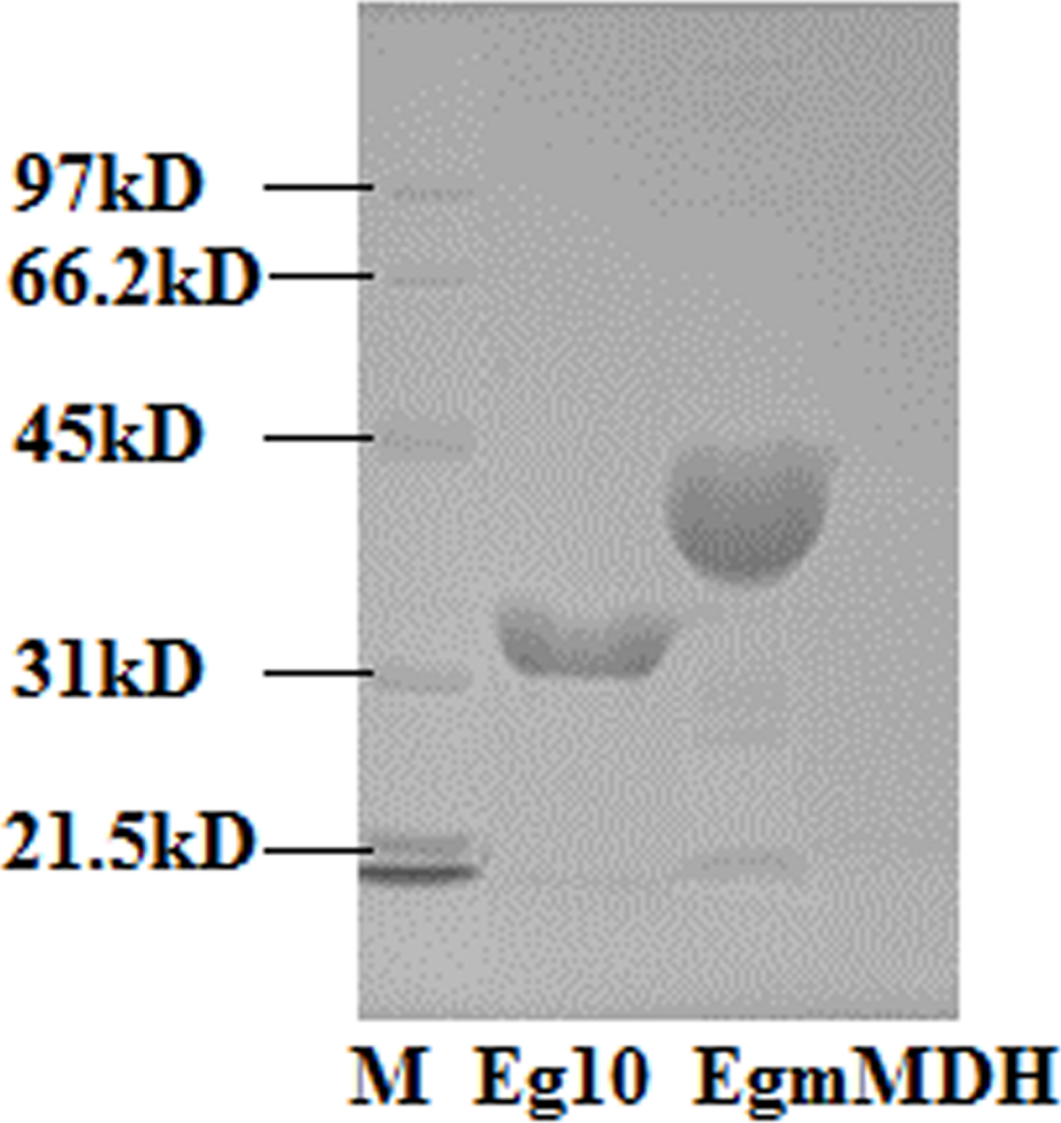
Eg10 and Eg mMDH were separated by 12% SDS-PAGE.

In SDS-PAGE analysis, Eg10 produced a band at ~31 kD and Eg mMDH at ~38 kD (Fig 1), they had higher purity and can be used do later experiment. The results of flow-cytometric analysis of the expression of DC-surface molecules on BMDCs of each treatment group in experiment (1) are presented in Table 1. The results showed that Eg10 upregulated the expression of MHCII and CD86 as compared with the control group (*P<*0.05), and slightly, albeit insignificantly, upregulated CD80 and CD40. Eg mMDH significantly upregulated CD86 and CD40 expression in the DCs as compared to the control and Eg10 treatments (*P<*0.05), and slightly, but insignificantly, upregulated CD80. Eg mMDH did not affect the expression of MCHII, while LPS treatment clearly stimulated the expression of DC-surface molecules MHCII and CD80 to significantly higher levels than those observed in the other three groups (*P<*0.05). In the presence of LPS, the expression levels of CD86 and CD40 were higher than those in the control and Eg10 groups, but not different from or even lower than those in the Eg mMDH group (*P*>0.05). LPS promoted Eg10-treated DCs to significantly upregulate the expression of MHCII and CD80 (*P*<0.05); however, the expression was still lower than that in LPS-treated DCs. However, LPS-stimulated DCs, when treated with Eg10, significantly downregulated the expression of CD80, CD86, and MHCII (*P*<0.05) (Table 2). Similarly, as shown in Table 3, LPS promoted Eg mMDH-treated DCs to significantly upregulate MHCII and CD80 expression and to downregulate CD40 expression (*P*<0.05). Like Eg10, Eg mMDH induced downregulation of CD80, CD86, CD40 and MHCII expression in LPS-stimulated DCs, (*P*<0.05). These results indicated that Eg10 and Eg mMDH can prevent the transformation of immature to mature DCs. To confirm the inference, morphology of DCs, phagocytic abilities of DCs and T cell proliferation induced by DCs also were detected.

**Table 1:** Surface molecular expression on DC treated with different antigen.

	CD80	MHCII	CD86	CD40
**control**	16.88±2.11	50.116±7.01	15.95±3.195	10.85±1.1
**Eg10**	18.71±3.36	56.67±2.41△&	27.73±3.57△	13.63±3.82
**mMDH**	20.58±2.45	49.78±1.76	36.2±2.07△#	20.99±3.05△#
**LPS**	49.5±3.85△#&	81.92±5.59△#&	34.38±2.917△#	16.28±4.79△

At 7 days, DCs at a concentration of 1×10^6^ cells/ml were incubated with Eg mMDH (1 µg/ml), Eg10 (1 µg/ml), or LPS (100 ng/ml) in RPMI-1640 culture medium in 24-well plates for 20 h and the surface markers were analyzed by flow cytometry. The results are expressed as the percentage of CD40-, CD80-, CD86-, and MHC-II- positive cells. Control means DCs are in RPMI1640 culture medium.

The P values as determined by Student’s paired t test for comparisons and the data are presented as means ± SEM of 7 independent experiments.

△*P*<0.05, significantly different from the control group.

# *P*<0.05, significantly different from the Eg10 group.

& *P*<0.05, significantly different from the Eg mMDH group.

**Table 2:** Compare to the surface molecules expression on DC treated with Eg10 plus LPS and DC treated with LPS plus Eg10.

	CD80	MHCII	CD86	CD40
**Eg10**	18.71±3.36	56.67±2.41	27.73±3.57	13.63±3.82
**LPS**	47.22±5.31 #●	77.71±4.27#●	38.35±1.35 #●▲	58.51±2.09#●▲
**LPS+Eg10**	20.74±1.92	61.67±5.33#	20.96±1.625 #	18.123±3.90#
**Eg10 +LPS**	42.57±2.31#	72.42±3.54#●	29.03±2.04●	16.23±1.02

DCs were incubated with Eg10 (1 µg/ml), in RPMI-1640 culture medium in 24-well plates for 20 h and with LPS (100 ng/ml) for 38 h. Then, LPS was added to the Eg10-treated DCs, while Eg10 were added to the LPS-treated DCs, after which the cells were incubated for another 18 h and the surface markers were analyzed by flow cytometry. The results are expressed as the percentage of CD40-, CD80-, CD86-, and MHC-II-positive cells.

The P values as determined by Student’s paired t test for comparisons and the data are presented as means ± SEM of 7 independent experiments.

**#** *P*<0.05, significantly different from the Eg10 group.

●*P*<0.05, significantly different from the LPS+Eg10 group.

▲*P*<0.05, significantly different from the Eg10+LPS group.

**Table 3:** Compare to the surface molecules expression on DC treated with Eg mMDH plus LPS and DC treated with LPS plus Eg mMDH.

	CD80	MHCII	CD86	CD40
**mMDH**	20.58±2.45	49.78±1.76	36.2±2.07▼	20.99±3.05
**LPS**	47.22±5.31&▼	77.71±4.27▼&	38.35±1.35▼	58.51±2.09▼&
**LPS+mMDH**	23.21±3.89	61.89±3.52 &	14.4±1.21	22.13±1.91
**mMDH +LPS**	49.55±1.05 ▼&	78.83±2.78 &▼	33.6±1.67▼	15.29±0.95

DCs were incubated with Eg mMDH (1 μg/ml), in RPMI-1640 culture medium in 24-well plates for 20 h and with LPS (100 ng/ml) for 38 h. Next, LPS was added to the Eg mMDH -treated DCs, while Eg mMDH were added to the LPS-treated DCs, after which the cells were incubated for another 18 h and the surface markers were analyzed by flow cytometry. The results are expressed as the percentage of CD40-, CD80-, CD86-, and MHC-II-positive cells.

The P values as determined by Student’s paired t test for comparisons and the data are presented as means ± SEM of 7 independent experiments.

& *P*<0.05, significantly different from the Eg mMDH group.

▼*P*<0.05, significantly different from the LPS+ Eg mMDH group.

*P*<0.05, significantly different from the Eg mMDH +LPS group.

### Phagocytic abilities of DCs exposed to antigens

**Fig 2:**
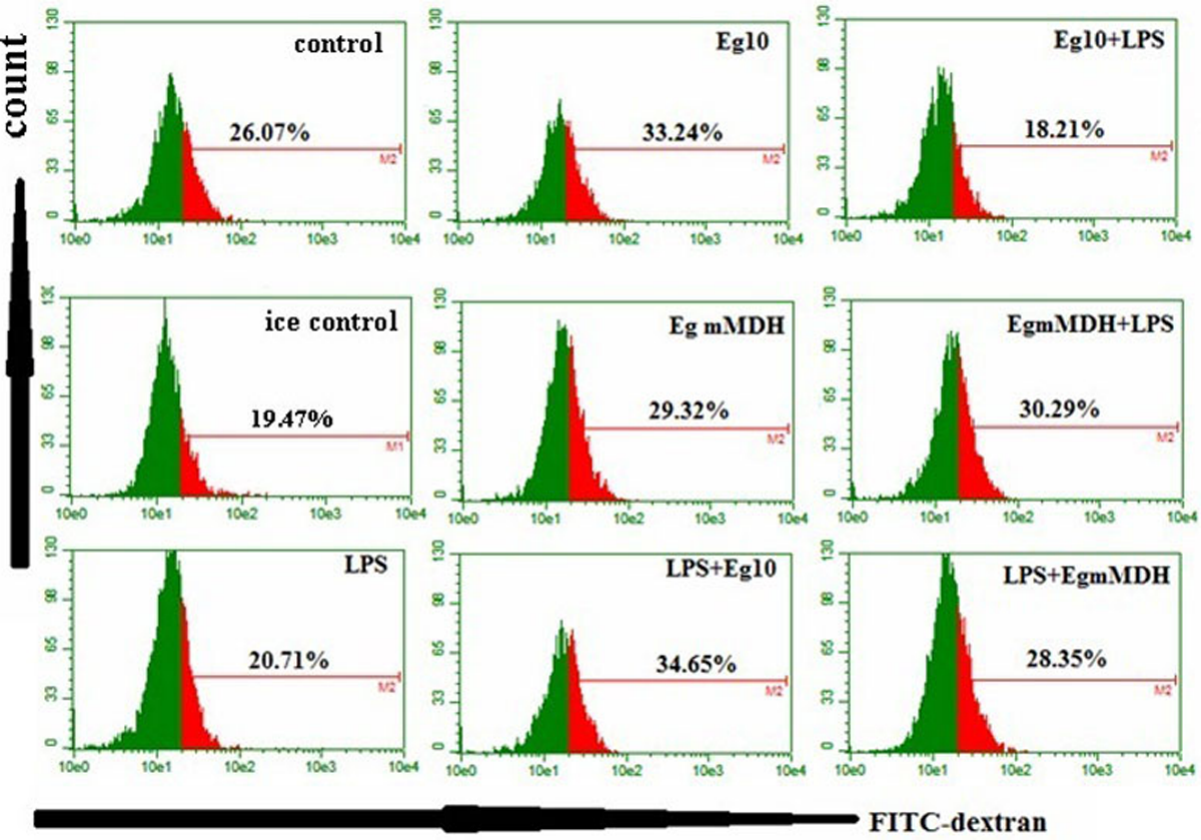
Phagocytic abilities of DCs exposed to antigens. BMDCs of each treatment group in experiment (1) were collected, and FITC-dextran (40 kDa) was added a final concentration of 1 mg/ml. The mixture was incubated at 37°C or at 4°C (for control binding)for 1 h and detected by flow cytometry. The uptake ability of DCs was measured by the intensity of fluorescence of FITC. Red shows the intensity of FITC of cells uptake FITC-dextran. Green shows the intensity of cell background. Each sample was analyzed in triplicate, and data were collected for 10,000 cells. Control means DCs are in RPMI1640 culture medium. Ice control is RPMI1640 culture medium treated at 4°C.

Measurements of the phagocytic ability indicated that LPS-treated DCs had reduced phagocytic abilities than those in other treatment groups except the Eg10+LPS group. Further, both Eg10 and Eg mMDH enhanced the ability of DCs to capture FITC-dextran. The addition of LPS resulted in a decrease in the capturing ability of the Eg10-treated DCs, while addition of LPS did not have an impact on the Eg mMDH-exposed DCs. However, the addition of Eg10 or Eg mMDH all increased the phagocytic ability of LPS-treated DCs. (Fig 2).

### Proliferative abilities of T cells cocultured with antigen-exposed DCs

**Fig 3:**
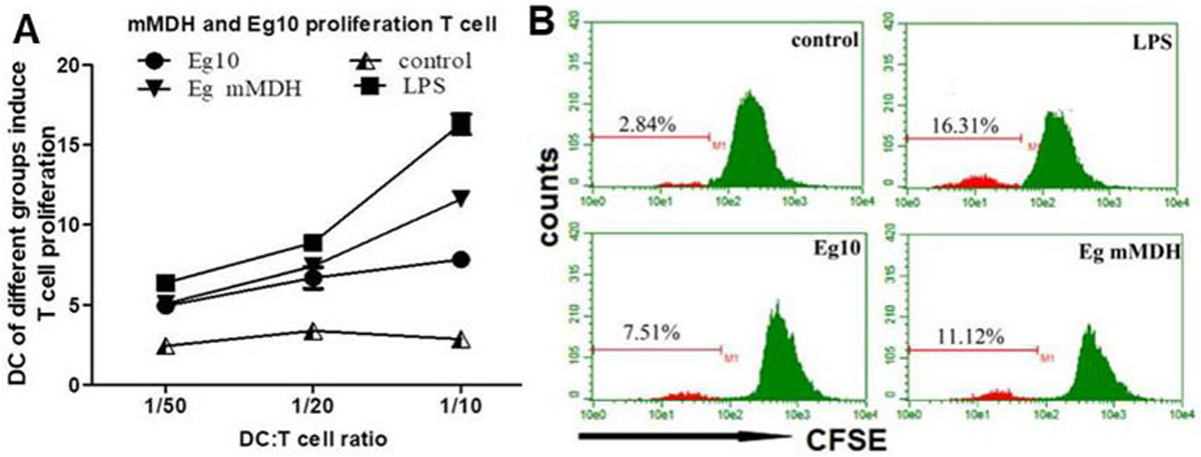
Proliferative abilities of T cells cocultured with antigen-exposed DCs. (A). DCs were activated with *Echinococcus granulosus* antigens and cocultured in graded amounts with allogeneic CD4^+^ T cells labeled with CFSE (DC/T cell ratio, 1:10, 1:20, 1:50). (B). Primary T cell proliferation was measured by determining the CFSE fluorescence intensity through flow cytometer. The data are means ± SD for triplicate cultures. Control means DCs are in RPMI1640 culture medium.

A comparison of the proliferative abilities of T cells induced by DCs of each treatment group indicated that the best proliferative ability was achieved when DCs were mixed with T cells at a ratio of 1:10(Fig 3A). LPS-treated DCs most strongly induced T-cell proliferation, followed by Eg mMDH-treated DCs, while Eg10-treated cells showed the weakest induction capacity (Fig 3B).

### Antigen-exposed DC morphology

**Fig 4:**
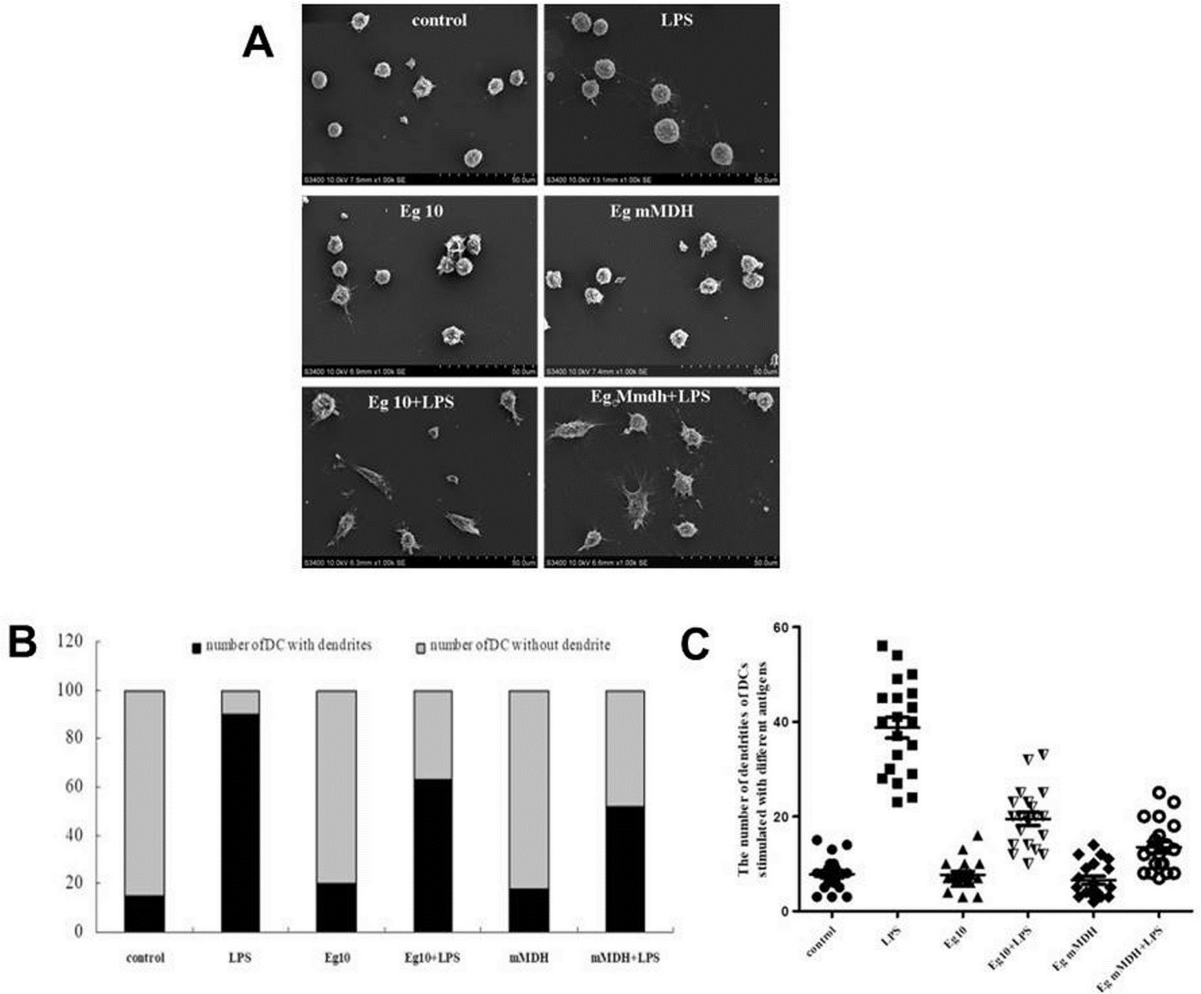
*Echinococcus granulosus* antigens effect on DC morphologic change. (A). DCs exposed to the various antigens were observed with a scanning electron microscope (SEM). (B). Quantification of the number of stimulated DCs that contain dendrites. Comparison of the ratio of DCs with or without dendrites among the treatment groups (100 DCs were counted randomly in each group). (C). DCs were cultured as above, and the number of dendrites of each DC was counted under SEM. The average dendrite number was compared between different groups. Bars represent means ± SEM (n = 20). Control means DCs are in RPMI1640 culture medium.

DCs exposed to the various antigens showed significantly different morphologies as observed by SEM. Control DCs had small volume and smooth surface with few creases and burr-like protrusions on the cell surface. The LPS-treated DCs had a large volume with more surface creases, and numerous thin and long extracellular protrusions, with the numbers of such protrusions being significantly higher than in each other treatment group (*P*<0.001)(Fig 4A). The DCs stimulated with antigen Eg10 or Eg mMDH alone showed similar morphology and surface protrusions as the control group; however, when LPS was added to these antigen-affected DCs, their morphologies changed, neither resembling those in the control group nor those in the LPS group. The antigen-affected DCs showed an increase in volume and became elongated, with some showing a fusiform-like shape and some resembling tadpoles, with an enlargement at one end and thinning on the other end (Fig 4A). Moreover, numerous short, thick protrusions were visible, with the number of protrusions being significantly higher in LPS add to the Eg10 and Eg mMDH groups than in the control group (*P*<0.001) as shown in Fig 4B. Further statistical analysis of the number of surface protrusions in each group of DCs suggested that although LPS could increase the number of protrusion-containing DCs in the Eg10 and Eg mMDH groups, the number of protrusions was still quite low, especially in the Eg mMDH group (Fig 4C).

### Expression of IDO in antigen-treated DCs

**Fig 5:**
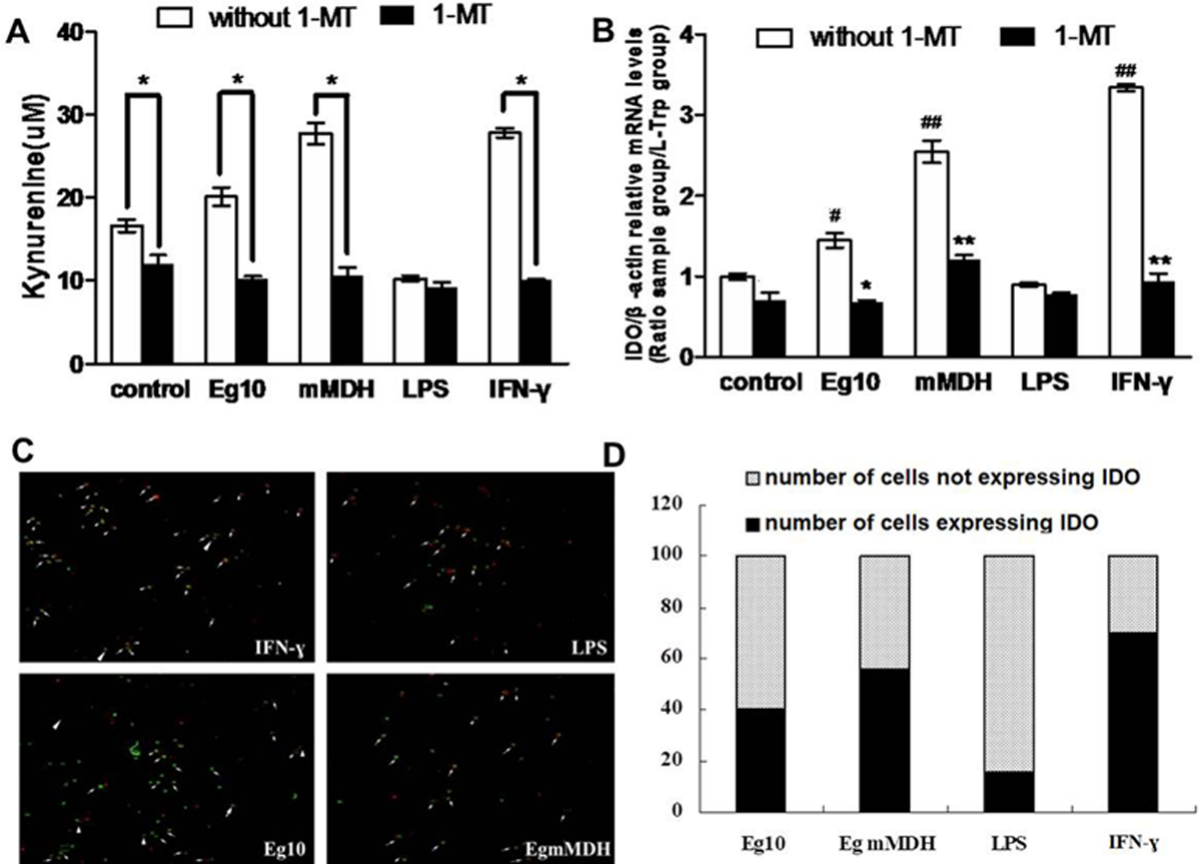
Expression of IDO in antigen-treated DCs. At 7d, each BMDC treatment group in experiment (2) was collected. (A). the kynurenine content in the supernatant was measured by ELISA. The numbers in Y-axis indicate the range of concentrations of kynurenine in the supernatant. Bars represent means ± SEM. (B). The mRNA content of IDO in each treatment group was detected by real time qPCR. The experiment was performed in triplicate and the mean values were reported. Bars represent means ± SEM. (C).Double label fluorescence immunohistochemistry staining and confocal laser scanning microscope showed IDO expression in different group. Under fluorescence microscopy, IDO was stained with red, the cell nucleus was stained as green with CFSE. The white arrows in the figures represent the cells expression IDO. (D). The ratio of IDO+DC/IDO–DC in different groups: ▓ indicates number of cells not expressing IDO; ◼indicates number of cells expressing IDO. Control means DCs are in RPMI1640 culture medium. ^#^ *P*<0.05 and ^##^ *P*<0.01, significantly different from the control group. *P<0.05 and **P<0.01, significantly different from the group in the absence of 1-MT.

Kynurenine is the primary product in the metabolic pathway of tryptophan, and its quantity reflects IDO activity. The detection of kynurenine content in the supernatant of each group of DCs revealed that Eg10- and Eg mMDH-treated DCs produced high concentrations of kynurenine, with the levels being significantly higher than that in the LPS group (*P*<0.05). After 3-h pretreatment with the IDO inhibitor 1-MT, the concentrations of kynurenine in each antigen-treated group showed a similar decrease (*P*<0.05), which was similar to the result in the IFN-γ group, while the kynurenine concentrations in the LPS group were very low both before and after the treatment (Fig 5A). In addition, qPCR was used to measure the IDO mRNA content in each group of DCs treated with or without 1-MT. The results indicated that the mRNA levels in the Eg10- and Eg mMDH-treated DCs were lower than that in the IFN-γ group, but significantly higher than that in the LPS group (*P*<0.05). Treatment with 1-MT significantly suppressed the increase in IDO mRNA in all groups except the LPS group (*P<0*.*05*) (Fig 5B). Detection of IDO expression by fluorescence immunohistochemistry (Fig 5C) revealed that the ratio of IDO^+^DC/IDO^–^DC in the LPS group was significantly lower than that in the IFN-γ group, while the expression levels in the Eg10 and Eg mMDH groups were between those in the IFN-γ and LPS groups (Fig 5D). These observations were consistent with the above results. Both Eg10 and Eg mMDH upregulated IDO expression in DCs, and especially, Eg mMDH had a stronger stimulatory effect than Eg10, indicating that Eg mMDH induced stronger IDO activity than Eg10.

### Production of Tregs in antigen-treated DC-stimulated T cells

**Fig 6:**
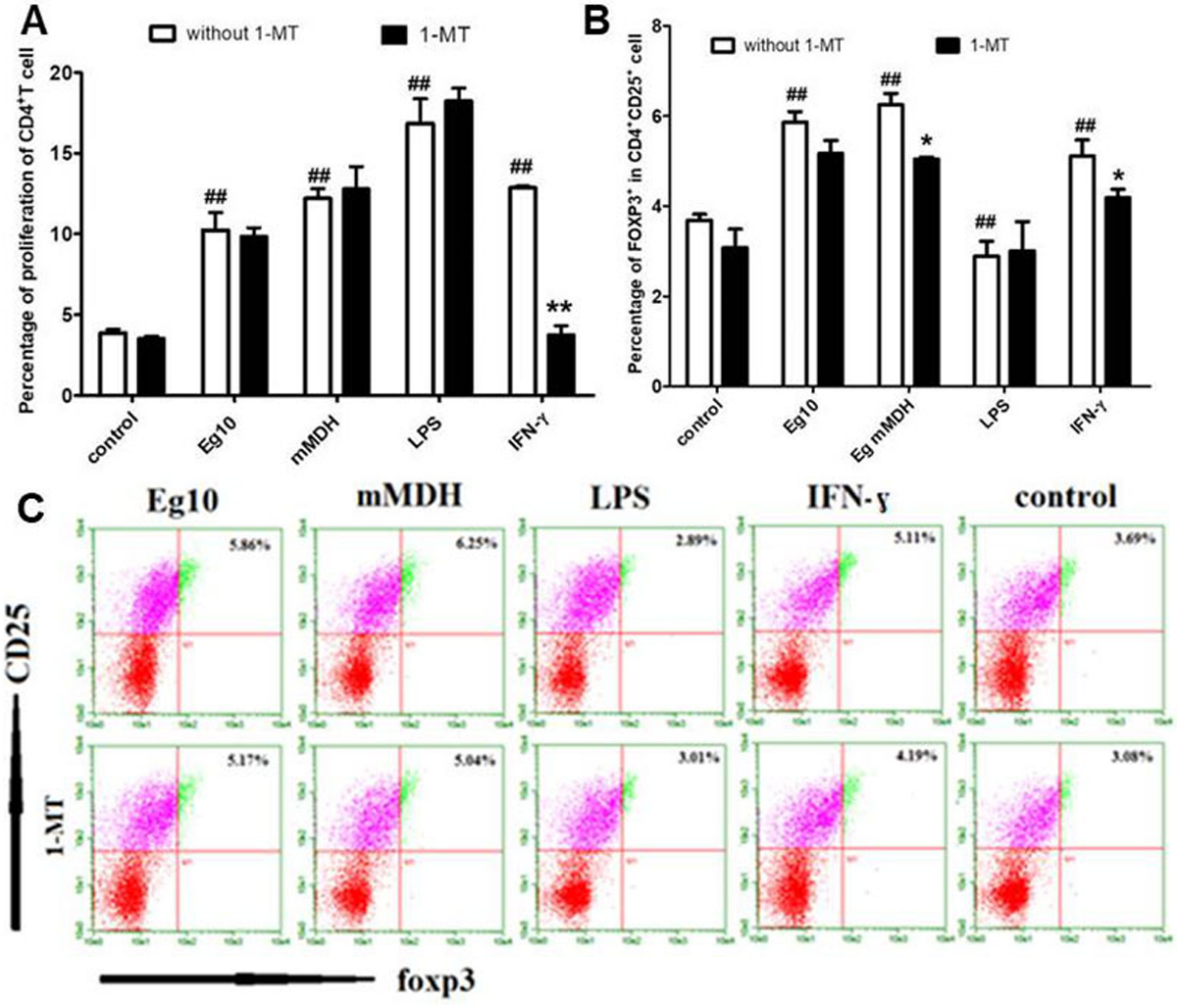
Formation of Tregs in antigen-treated DC-stimulated T cells. **(**A). DCs were stimulated with different *Echinococcus granulosus* antigens in the presence or absence of the IDO inhibitor 1-MT and co-cultured with T cells (MLR). (B) and (C). At the end of the co-culture T cells of different groups were collected and examined for CD25 and foxp3 expression by flow cytometry respectively. The data are presented as means ± SEM of 4 independent experiments. Control means DCs are in RPMI1640 culture medium. ^#^ *P*<0.05 and ^##^ *P*<0.01, significantly different from the control group. *P<0.05 and **P<0.01, significantly different from the group in the absence of 1-MT.

In this experiment, DCs treated with antigens were cocultured with CD4^+^ T cells that were isolated by magnetic beads and labeled with CFSE in a ratio of 1:10. Flow-cytometric analysis indicated that compared with the LPS group, the two antigen-exposed groups and the IFN-γ group induced lower levels of CD4^+^ T-cell proliferation (*P*<0.05). In cells pretreated with 1-MT, only the Eg mMDH group induced an elevated level of CD4^+^ T-cell proliferation (*P*<0.05), while the IFN-γ group showed a decreased level of CD4^+^ T-cell proliferation (*P*<0.05) and the Eg10 group did not show an obvious change (*P*>0.05) (Fig 6A). These results indicated that IDO might play a role in Eg mMDH effectively enhance the ability of DCs to induce T-cell proliferation, while it does not seem to affect the same process in cells exposed to Eg10, likely due to the presence of other, unknown mechanisms. Intracellular foxp3 staining was employed to detect the number of CD4^+^CD25^+^foxp3^+^ Tregs. The number of CD4^+^CD25^+^foxp3^+^ Tregs was high in the two antigen-treated groups and in the IFN-γ group, and 1-MT treatment significantly decreased the amount in the Eg mMDH and IFN-γ groups (*P*<0.05) and slightly but insignificantly in the Eg10 (*P*>0.05), as shown in Fig 6B and 6C.

### Effect of the antigens on Th1/Th2 cytokines

**Fig 7:**
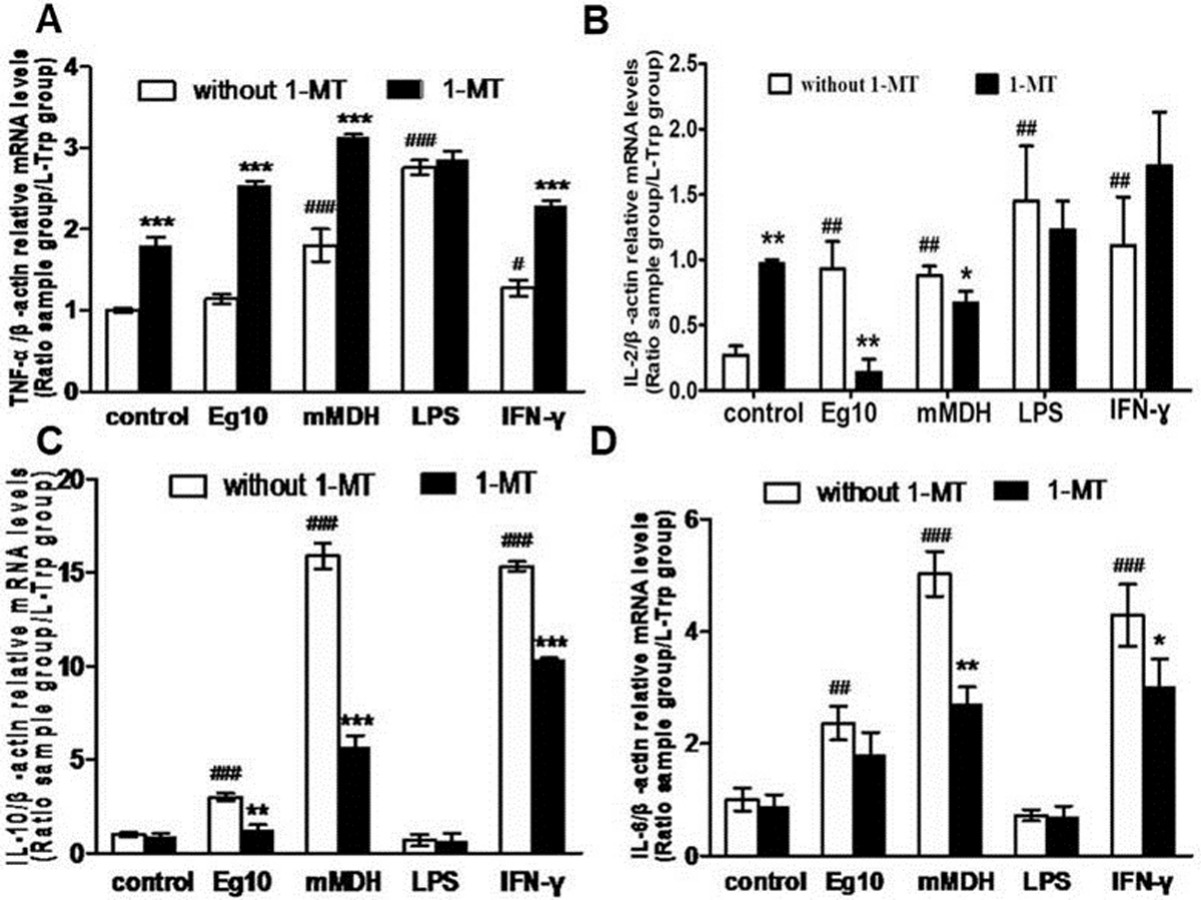
mRNA expression of cytokines DCs effected by *Echinococcus granulosus* antigens. DCs from each treatment group in experiment (2) were collected and detected the mRNA level of different cytokines. Control means DCs are treated with RPMI1640. (A). the mRNA level of TNF-α. (B). the mRNA level of IL-2. (C). the mRNA level of IL-6. (D). the mRNA level of IL-10. ^#^ *P*<0.05, ^##^ *P*<0.01 and ^###^ *P*<0.001, significantly different from the control group. *P<0.05, **P<0.01 and ***P<0.001, significantly different from the group in the absence of 1-MT.

**Fig 8:**
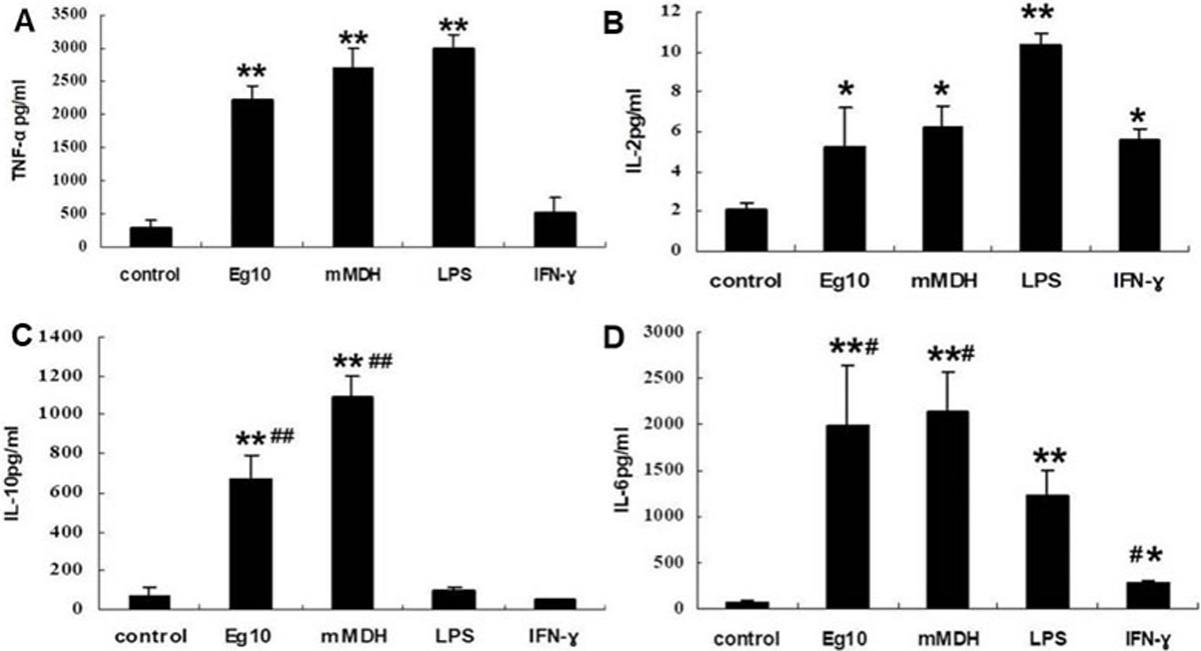
cytokines of different groups of DCs culture supernatant from each treatment group in experiment (2) were collected and detected the level of different cytokines. Control means DCs are treated with RPMI1640. (A). the level of TNF-ɑ of supernatant (B). the level of IL-2 of supernatant (C).the level of IL-10 of supernatant (D). the level of IL-6 of supernatant. *P<0.05, **P<0.001 significantly different from the control group. ^#^ *P*<0.05, ^##^ *P*<0.001 significantly different from the LPS group.

The mRNA expression levels of TNF-α, IL-2, IL-6, and IL-10 in each group of DCs were measured by qPCR. Compared with the control group, the Eg mMDH, LPS, and IFN-γ groups showed elevated levels of TNF-α expression (*P*<0.05), while each of the groups treated with 1-MT, except for the LPS group, showed significantly elevated expression of TNF-α (*P*<0.05) (Fig 7A). IL-2 expression was higher in each group of treated DCs than in the control group, and after 1-MT treatment, only the control and IFN-γ groups showed elevated IL-2 expression, while the secretion levels were decreased in the Eg10, Eg mMDH, and LPS groups (Fig 7B). IL-6 and IL-10 mRNA expression was significantly elevated in the Eg10 and IFN-γ groups (*P*<0.05), especially, in the Eg mMDH group (*P*<0.001). 1-MT suppressed the expression of IL-6 and IL-10 expression to a certain degree in the three groups, while LPS-treated cells consistently showed low levels of expression, with or without 1-MT (Fig 7B and 7C). And partly same results were observed in detection of cytokines of different DCs groups’ culture supernatant. Eg10, Eg mMDH and LPS all induced DCs to secrete higher level of TNF-α than control group. (*P<0*.*001*), but there was no significant distinct between them(Fig 8A). And all groups can secrete higher level of IL-2 than control group, especially LPS which level was about twofold than Eg10, EgmMDH and IFN-γ(Fig 8B). Eg10, Eg mMDH induced DCs production dramatic IL-10 and IL-6 than LPS, IFN-γ and control groups(*P<0*.*001*) (Fig 8C and 8D). In brief, Eg10 and Eg mMDH induced DCs expression and secretion higher IL-6 and IL-10, especially for Eg mMDH which induce DCs expression mRNA level of IL-6 and Il-10 can be decreaed by 1-MT. The results indicated that IDO maybe played a role in Eg mMDH induced DCs immaturation.

## Discussion

Eg10 is a hydrophilic protein antigen expressed on the surface of *E*. *granulosus* protoscoleces and may be associated with the growth and metabolism of the worm [13]. Eg mMDH, a type of malate dehydrogenase with high activity, is primarily present in the cytoplasm and mitochondria of protoscoleces [14], where it participates in intracellular material transfer and plays an important role in gluconeogenesis and glycolysis. Given that the two antigens play an important role in protoscolex growth and metabolism and have potential as hydatid vaccine targets, we tested infection resistance in the immunized mice, and the results indicated that the immunization not only failed to increase the resistance to infection by *E*. *granulosus* protoscolex, but even aggravated the infection compared with the control group [15,16]. This observation was quite intriguing and raised the question as to which immune reactions occurred in the mice after immunization with the two antigens to allow the protoscolex to escape from immune attack.

A study by Diaz suggested that the antigen AgB of *E*. *granulosus* may cause different maturity of DCs by inhibiting the differentiation of monocytes to DCs or affecting DC maturation, and that the different maturity may affect the abilities of DCs for antigen presentation, inducing T-cell proliferation, and inducing immune reactions [17]. Our results were consistent with those findings, in that Eg10 and Eg mMDH significantly inhibited MCHII expression on DCs, while the expression of CD80 was not significantly affected, that of CD86 was promoted by the two antigens(Table 1), especially by Eg mMDH which slightly enhanced CD40 expression. Eg10 and Eg mMDH led to strong antigen-capturing ability, while having a weak induced effect on T-cell proliferation (Figs 2 and 3). It is well known that costimulatory signals are pivotal for T-cell activation, with a lack of either receptor or ligands likely to severely disrupt T-cell activity so as to induce tolerance [18]. There has been controversy over the increased expression of CD86, and it is currently accepted that CD80 and CD86 of DCs regulate Th1 and Th2 reactions, respectively, and transduce various signals to helper T cells, thereby inducing the differentiation of Th0 cells to different Th cells and inducing different immune reactions [19]. Findings similar to ours" here was reported by Rigano et al. [20], who conducted experiments to investigate the effects of AgB and SHF on DCs. Casaravilla et al. [21] suggested that this was associated with the characteristics of semimature DCs.

LPS significantly increased the expression of MHCII and CD80 in the two groups of DCs, while Eg10 and Eg mMDH inhibited the expression of MHCII, CD80, and CD86, which indicated that the two antigens were unable to effectively stimulate DCs to undergo maturation, leaving them in a semimature state (Table 1). In addition, the semimature status affected the phagocytic activity of DCs, which was experimentally assessed by detecting the antigen-capturing ability of DCs. Further, these results were supported by SEM observation of cell morphology. Protrusions on the surface of DCs have special functions, including establishing a good contact between DCs and T cells [22]. The number of protrusions may reflect the degree of DCs maturity and their antigen-presentation ability, and is also associated with the numbers and proliferative abilities of T cells activated by DCs, thereby affecting the proliferation of T-cell subsets and regulating the relevant immune responses [23]. Our study indicated that after the addition of LPS, Eg10- and Eg mMDH-treated DCs were more matured and showed a significant increase in burr-like protrusions, and changed from circular to fusiform or tadpole-shaped morphology (Fig 4). This implies that the DCs were in a semi-immature status in which the maturity was slightly elevated, the antigen-presentation ability was weakened, and the ability to induce T-cell proliferation was enhanced to some extent but still quite weak. The morphological findings were consistent with those reported by Ferret-Bernard et al[24]. (Who conducted a proteomics study on the effect of antigens of intestinal parasites on DCs; when interacted with different antigens, DCs showed different morphologies due to a change in skeleton proteins. This change affected DC functions and especially, mobility for capturing antigens, with the antigen-capturing mobility function being promoted by a bipolar, spindle-like morphology. When DCs develop from the earliest stage of immaturity to a mature stage under the interaction of antigens, they change from a bipolar, spindle-like shape to a tadpole-like shape and eventually, to a circular morphology of complete maturity without pseudopods. DCs with transient morphologies are neither completely mature nor immature, and this status is referred to as restrictive maturity or semimaturity. Semimature DCs are able to induce Th2 immune reactions. It was speculated that the two antigens promoted DCs to secrete cytokines IL-10 and IL-6 to inhibit DCs from inducing T-cell proliferation [25]. In this study, qPCR and cytokines in culture supernatant analyses indicated that Eg10 and Eg mMDH induced high IL-6 and IL-10 expression but low IL-2 expression (Figs 7 and 8). IL-6 promotes DCs to enter a semimature status [26].

High expression of IDO in DCs reportedly increased leishmaniasis infection and facilitated pathogen survival as well [9]. IDO inhibits T-cell proliferation by depleting tryptophan [27,28], and the product kynurenine induces T-cell anergy while increasing the fraction of Treg cells, indicating IDO plays an important role in immune tolerance [29]. The results in this study were consistent herewith. Upon stimulation of DCs with Eg10 and Eg mMDH, IDO mRNA expression and kynurenine secretion were significantly elevated. The expression level in the Eg mMDH group was not significantly different from that in the positive control group IFN-γ. Similar results were obtained with cell fluorescence immunohistochemistry (Fig 5).

By using a CD4^+^ T-cell knockout mouse model, Baz et al. [30] verified that in the early stage of infection by *E*. *granulosus* protoscoleces, the infection was primarily resisted by proliferating CD4^+^ T cells, while the absence of effective effector T cells led to chronic infection and promoted parasite survival. In a previous study by our research group, we evaluated spleen lymphocyte subsets in Eg10- and Eg mMDH-immunized mice that were infected with protoscoleces[15,16]. We found that there was no significant difference in the content of CD4^+^ T cells between immunized and control mice. In the current study, we found that the two antigens acted on DCs to induce weak proliferation of CD4^+^ T cells, suggesting that this was the primary reason for mice immunized with the two antigens can not be against infected by the protoscolex. Moreover, high expression of CD4^+^ CD25^+^ foxp3^+^ was detected, and Eg mMDH promoted the formation of Treg cells more strongly than IFN-γ, with 1-MT inhibiting the increase in Tregs (Fig 6). Tregs primarily play a role in immune tolerance by inhibiting inherent as well as acquired immunity [31], disrupting host immune function and leading to a massive secretion of the inhibitory inflammatory cytokine IL-10 as well as inhibiting the generation of the Th1 cytokines IL-2 [25,32]. This may explain the aggravated infection in Eg mMDH-immunized mice (Fig 8), with the aggravated infection likely involving an IDO-dependent immune escape mechanism.

In summary, the mechanism of aggravated infection of Eg mMDH-immunized mice with *E*. *granulosus* protoscolex may involve two processes. On the one hand, Eg mMDH inhibits DCs from developing to full maturity, resulting in a semimature status. Given the low expression of costimulatory molecules in such DCs, DCs have a weak ability to stimulate the proliferation of CD4^+^ T cells and preferentially induce a Th2 immune response. On the other hand, the semimature DCs strongly express IDO and deplete tryptophan, and they inhibit T-cell proliferation while promoting Treg generation, thus causing T-cell anergy and promoting IL-10 secretion while suppressing Th1 cytokines, enabling immune escape. The escape process could be inhibited by the IDO inhibitor 1-MT, and was therefore referred to as an IDO-dependent immune escape mechanism. The addition of Eg10 aggravated the infection, via another mechanism. Eg10 promoted DCs to develop semimaturity, led to low expression of costimulatory molecules, and inhibited T-cell proliferation, but we were unable to prove that the Eg10-aggravated infection was attributable to IDO. Obviously, the way *E*. *granulosus* escapes from immune surveillance and attack is not unique and there might exist other immune mechanisms. However, the “IDO-dependent immune escape” hypothesis can aid in gaining a better understanding of the mechanisms of immune tolerance after entry of *E*. *granulosus* into the body and lays a foundation for further studying the immune pathogenesis of hybrid diseases and exploring new immunotherapy targets.
